# Supplementary material for: Polyketide synthase gene clusters in the ericoid mycorrhizal fungus Oidiodendron maius: genome-wide prediction, expression profiling, and functional characterization of knockout mutants
Source: Front Plant Sci. 2026 Sep 16;17:1924430. doi: 10.3389/fpls.2026.1924430 (PMC13623903; doi:10.3389/fpls.2026.1924430)
Supplement: Supplementary file 1 [file SupplementaryFile1.zip › Supplementary Materials/Supplementary Folder 1.zip!/Oidma1_AssemblyScaffolds_Repeatmasked/knownclusterblast/cluster13/jgi.p_Oidma1_51158_mibig_hits.html]

| MIBiG Protein | Description | MIBiG Cluster | MiBiG Product | % ID | % Coverage | BLAST Score | E-value |
| --- | --- | --- | --- | --- | --- | --- | --- |
| AAK33080.1 | putative\_trichothecene\_C-15\_hydroxylase | BGC0001277 | Nivalenol\_/\_deoxynivalenol\_/\_3-acetyldeoxynivalenol\_/ | 54 | 94.9019607843 | 545 | 6.2e-155 |
| AAK53587.1 | isotrichodermin\_C-15\_hydroxylase | BGC0000931 | Trichothecene\_biosynthetic\_gene\_cluster | 54 | 94.9019607843 | 543 | 2.4e-154 |
| AAK33070.1 | trichothecene\_C-15\_hydroxylase | BGC0001278 | Nivalenol\_/\_deoxynivalenol\_/\_3-acetyldeoxynivalenol\_/ | 54 | 94.9019607843 | 541 | 9e-154 |
| AAK53580.1 | isotrichodermin\_C-15\_hydroxylase | BGC0000930 | Trichothecene\_biosynthetic\_gene\_cluster | 53 | 94.9019607843 | 535 | 4.9e-152 |
| ACD39751.1 | cytochrome\_P450 | BGC0000076 | Hypothemycin\_biosynthetic\_gene\_cluster | 41 | 92.9411764706 | 397 | 2.7e-110 |
| ACD39760.1 | cytochrome\_P450 | BGC0000077 | Hypothemycin\_biosynthetic\_gene\_cluster | 41 | 92.9411764706 | 396 | 6.1e-110 |
| BAE56591.1 |  | BGC0001123 | Aspirochlorine\_biosynthetic\_gene\_cluster | 42 | 94.9019607843 | 370 | 3.6e-102 |
| OAQ83755.1 | isotrichodermin\_C-15\_hydroxylase | BGC0001358 | Leucinostatins\_biosynthetic\_gene\_cluster | 40 | 97.4509803922 | 365 | 1.2e-100 |
| ACH72905.1 | AflG | BGC0000011 | Aflatoxin/sterigmatocystin\_biosynthetic\_gene\_cluster | 41 | 97.0588235294 | 354 | 1.6e-97 |
| AAS90058.1 | AvnA | BGC0000009 | Aflatoxin\_biosynthetic\_gene\_cluster | 40 | 92.9411764706 | 353 | 4.5e-97 |
| AAS90078.1 | AvnA | BGC0000010 | Aflatoxin\_biosynthetic\_gene\_cluster | 40 | 92.9411764706 | 351 | 2.2e-96 |
| AAS90010.1 | AvnA | BGC0000007 | Aflatoxin\_biosynthetic\_gene\_cluster | 39 | 96.0784313725 | 349 | 8.5e-96 |
| AAS90101.1 | AvnA | BGC0000006 | Aflatoxin\_biosynthetic\_gene\_cluster | 40 | 92.9411764706 | 348 | 1.1e-95 |
| AAS90032.1 | AvnA | BGC0000008 | Aflatoxin\_biosynthetic\_gene\_cluster | 40 | 92.9411764706 | 347 | 1.9e-95 |
| BAE71325.1 | cytochrome\_P450\_monooxygenase | BGC0000004 | Aflatoxin\_biosynthetic\_gene\_cluster | 39 | 92.9411764706 | 343 | 3.6e-94 |
| BAQ25465.1 | cytochrome\_P450 | BGC0001280 | Betaenone\_C\_/\_betaenone\_A\_biosynthetic\_gene\_cluster | 39 | 95.4901960784 | 339 | 5.2e-93 |
| AAC49196.1 | putative\_p450\_monooxygenase | BGC0000152 | Sterigmatocystin\_biosynthetic\_gene\_cluster | 41 | 91.568627451 | 338 | 1.2e-92 |
| BAK26559.1 | cytochrome\_P450 | BGC0000977 | Cyclopiazonic\_acid\_biosynthetic\_gene\_cluster | 38 | 97.0588235294 | 329 | 9.2e-90 |
| BAD29972.1 | P450\_monooxygenase\_1 | BGC0000676 | Aphidicolin\_biosynthetic\_gene\_cluster | 38 | 95.2941176471 | 324 | 1.7e-88 |
| AAS90059.1 | VerB | BGC0000009 | Aflatoxin\_biosynthetic\_gene\_cluster | 38 | 96.6666666667 | 320 | 3.3e-87 |
| AAC49200.1 | putative\_p450\_monooxygenase | BGC0000152 | Sterigmatocystin\_biosynthetic\_gene\_cluster | 37 | 94.7058823529 | 320 | 4.3e-87 |
| AAS90011.1 | VerB | BGC0000007 | Aflatoxin\_biosynthetic\_gene\_cluster | 37 | 97.4509803922 | 319 | 7.3e-87 |
| AAS90102.1 | VerB | BGC0000006 | Aflatoxin\_biosynthetic\_gene\_cluster | 37 | 96.6666666667 | 317 | 3.6e-86 |
| AAS90079.1 | VerB | BGC0000010 | Aflatoxin\_biosynthetic\_gene\_cluster | 37 | 96.6666666667 | 317 | 3.6e-86 |
| BAE71326.1 | desaturase | BGC0000004 | Aflatoxin\_biosynthetic\_gene\_cluster | 37 | 96.6666666667 | 316 | 8e-86 |
| AAS90033.1 | VerB | BGC0000008 | Aflatoxin\_biosynthetic\_gene\_cluster | 37 | 96.6666666667 | 315 | 1e-85 |
| XP\_002384857.1 | cytochrome\_P450,\_putative | BGC0001234\_c2 | 4,4'-piperazine-2,5-diyldimethyl-bis-phenol\_biosynthetic\_gene | 36 | 99.0196078431 | 310 | 3.4e-84 |
| ACB12551.1 | Fum2 | BGC0000063 | Fumonisin\_biosynthetic\_gene\_cluster | 34 | 97.2549019608 | 305 | 1.4e-82 |
| ACD39773.1 | cytochrome\_P450 | BGC0000134 | Radicicol\_biosynthetic\_gene\_cluster | 36 | 96.0784313725 | 301 | 2e-81 |
| AAN74815.2 | Fum2p | BGC0000062 | Fumonisin\_biosynthetic\_gene\_cluster | 34 | 98.8235294118 | 298 | 1.3e-80 |
| AAM18769.1 | AflL | BGC0000011 | Aflatoxin/sterigmatocystin\_biosynthetic\_gene\_cluster | 35 | 96.862745098 | 292 | 1.2e-78 |
| Bcin12g06370.1 |  | BGC0000631 | Botrydial\_biosynthetic\_gene\_cluster | 35 | 90.3921568627 | 290 | 2.8e-78 |
| EFY94501.1 | benzoate\_4-monooxygenase\_cytochrome\_P450 | BGC0000337 | Destruxins\_biosynthetic\_gene\_cluster | 33 | 101.568627451 | 275 | 9.2e-74 |
| ADI24937.1 | VrtK | BGC0000168 | Viridicatumtoxin\_biosynthetic\_gene\_cluster | 35 | 98.2352941176 | 275 | 1.2e-73 |
| BAV32150.1 | cytochrome\_P450\_monooxygenase | BGC0001373 | Sordarin\_biosynthetic\_gene\_cluster | 32 | 98.0392156863 | 255 | 9.8e-68 |
| BAD83680.1 | cytochrome\_P-450 | BGC0000012 | Alternapyrone\_biosynthetic\_gene\_cluster | 33 | 101.37254902 | 255 | 1.3e-67 |
| AGC83578.1 | P450\_monooxygenase | BGC0000818 | Notoamide\_biosynthetic\_gene\_cluster | 28 | 86.6666666667 | 183 | 6.2e-46 |
| BAH23996.1 | cytochrome\_P450 | BGC0000356 | Fumitremorgin\_biosynthetic\_gene\_cluster | 29 | 91.7647058824 | 183 | 6.2e-46 |
| ADM34140.1 | P450 | BGC0001084 | Notoamide\_/\_stephacidin\_biosynthetic\_gene\_cluster | 28 | 94.9019607843 | 181 | 1.8e-45 |
| OAQ83761.1 | cytochrome\_P450 | BGC0001358 | Leucinostatins\_biosynthetic\_gene\_cluster | 30 | 59.8039215686 | 147 | 3.8e-35 |
| AGA37280.1 | P450\_monooxygenase | BGC0000819 | Paraherquamide\_biosynthetic\_gene\_cluster | 28 | 89.2156862745 | 141 | 3.5e-33 |
| ctg1\_orf003 |  | BGC0000687 | Phyllocladan-16alpha-ol\_biosynthetic\_gene\_cluster | 43 | 24.9019607843 | 107 | 4.3e-23 |
| AAS90057.1 | VerA | BGC0000009 | Aflatoxin\_biosynthetic\_gene\_cluster | 25 | 74.3137254902 | 106 | 7.4e-23 |
| ACH72911.1 | AflV | BGC0000011 | Aflatoxin/sterigmatocystin\_biosynthetic\_gene\_cluster | 24 | 85.2941176471 | 105 | 2.1e-22 |
| AIA58896.1 | putative\_cytochrome\_P450 | BGC0001141 | Brefeldin\_biosynthetic\_gene\_cluster | 25 | 95.2941176471 | 105 | 2.1e-22 |
| ACH72913.2 | AflN | BGC0000011 | Aflatoxin/sterigmatocystin\_biosynthetic\_gene\_cluster | 33 | 38.431372549 | 99 | 1.2e-20 |
| ctg1\_orf5 |  | BGC0000687 | Phyllocladan-16alpha-ol\_biosynthetic\_gene\_cluster | 38 | 27.2549019608 | 83 | 8.7e-16 |
